# ABT-263 induces apoptosis and synergizes with chemotherapy by targeting stemness pathways in esophageal cancer

**DOI:** 10.18632/oncotarget.4540

**Published:** 2015-07-17

**Authors:** Qiongrong Chen, Shumei Song, Shaozhong Wei, Bin Liu, Soichiro Honjo, Ailing Scott, Jiankang Jin, Lang Ma, Haitao Zhu, Heath D. Skinner, Randy L. Johnson, Jaffer A. Ajani

**Affiliations:** ^1^ Departments of Gastrointestinal Medical Oncology, University of Texas MD Anderson Cancer Center, Houston, Texas 77030, USA; ^2^ Departments of Biochemistry & Molecular Biology, University of Texas MD Anderson Cancer Center, Houston, Texas 77030, USA; ^3^ Hubei Cancer Hospital, Wuhan 430079, China; ^4^ Departments of Genetics, University of Texas MD Anderson Cancer Center, Houston, Texas 77030, USA

**Keywords:** esophageal cancer, stemness pathways, cancer stem cells, ABT-263, 5-fluorouracil

## Abstract

Activation of cancer stem cell signaling is central to acquired resistance to therapy in esophageal cancer (EC). ABT-263, a potent Bcl-2 family inhibitor, is active against many tumor types. However, effect of ABT-263 on EC cells and their resistant counterparts are unknown. Here we report that ABT-263 inhibited cell proliferation and induced apoptosis in human EC cells and their chemo-resistant counterparts. The combination of ABT-263 with 5-FU had synergistic lethal effects and amplified apoptosis that does not depend fully on its inhibition of BCL-2 family proteins in EC cells. To further explore the novel mechanisms of ABT-263, proteomic array (RPPAs) were performed and gene set enriched analysis demonstrated that ABT-263 suppresses the expression of many oncogenes including genes that govern stemness pathways. Immunoblotting and immunofluorescence further confirmed reduction in protein expression and transcription in Wnt/β-catenin and YAP/SOX9 axes. Furthermore, ABT263 strongly suppresses cancer stem cell properties in EC cells and the combination of ABT-263 and 5-FU significantly reduced tumor growth *in vivo* and suppresses the expression of stemness genes. Thus, our findings demonstrated a novel mechanism of ABT-263 antitumor effect in EC and indicating that combination of ABT-263 with cytotoxic drugs is worthy of pursuit in patients with EC.

## INTRODUCTION

Esophageal carcinoma (EC) is a lethal disease with high incidence globally, and the incidence has been increasing in recent years [1]. Generally, the localized EC is treated with chemo-radiation therapy plus surgery, however, > 70% of patients have residual cancer in the surgical specimen and their prognosis remains poor [2–4]. This inherent resistance in EC is most likely due to the heterogeneity in the genetic makeup and large number of DNA alterations. The results from the current therapies are often devastating to the patient and family. In depth understanding of molecular oncology could improve therapeutic approaches. Many studies have indicated that overexpression of Bcl-2 family proteins is associated with tumor maintenance, metastatic progression, and therapy resistance [5, 6]. Accordingly, Bcl-2 family proteins can also act as the diagnostic and prognostic markers, but especially, as novel therapeutic targets [7, 8].

ABT-263, a new BH3 mimetic, is a potent Bcl-2 family inhibitor that antagonizes Bcl-2 family members (Bcl-2, Bcl-xL and Bcl-w) [9]. It was found safe and effective against some leukemia, lymphoma, small cell lung cancer, and other malignancies [10–14]. Nevertheless, in some xenograft models of aggressive lymphoma and in phase I/II clinic studies in patients of lung cancer, single-agent of ABT-263 just exhibited modest or limited efficacy [10, 11, 15] with dose-dependent thrombocytopenia induced by targeting Bcl-xL in megakaryocytes [10, 16]. Recently, the studies are focused on the combination of ABT-263 with cytotoxics. Ackler et al. found that ABT-263 enhances the response of multiple chemotherapeutic regimens in hematologic tumors [14]. The study from the same group found that ABT-263 and rapamycin act cooperatively to kill lymphoma cells *in vitro* and *in vivo* [17]. However, the effects of ABT-263 and in combination of chemotherapy and its mechanism of action have not been explored in EC.

Many studies suggest that a small subpopulation of cancer stem cells (CSCs) has the capacity to repopulate tumors and drive malignant progression and mediate radio- and chemoresistance [18]. Dysregulation of CSC signaling like Hippo/YAP1, Wnt/β-catenin, and hedgehog (Hh) have been implicated in the maintenance of tumor and in conferring therapy resistance [19–22]. We have previously reported that Hh pathway is often up-regulated in EC and mediates therapy resistance [23–25]. Yes-associated protein (YAP-1) is the downstream effector of the Hippo signaling pathway, which is frequently overexpressed in many types of cancers [26, 27]. Our recent studies have identified YAP-1 is a major inducer of CSC properties in non-tumorigenic cells as well as in EC cells by direct up-regulation of SOX9. Thus, the YAP-1-SOX9 axis could be an important therapeutic target in EC [20, 28]. Further, we also observed that YAP-1 mediates constitutive and acquired treatment resistance in EC cells [22]. Therefore, an agent that can block YAP-1/SOX9 expression or activity will be important in improving patient outcome.

5-FU is an old anti-cancer agent [29] and it is used frequently against EC [3, 29]. It has, however, limited cytotoxic activity [30–33]. However, if 5-FU can synergize with a targeted agent, it could provide a unique advantage. Thus we explored the effects of ABT-263 alone or combined with 5-FU on a variety of EC cell lines and demonstrated that ABT-263 with 5-FU synergistically enhances the sensitivity and bolsters apoptosis in EC cells and their therapy resistant counterparts. In addition, novel mechanisms of action of ABT-263 with cytotoxics on EC cells were explored.

## RESULTS

### ABT-263 inhibits EC cell growth and synergizes with 5-FU on both sensitive and resistant EC cells

**Figure 1:**
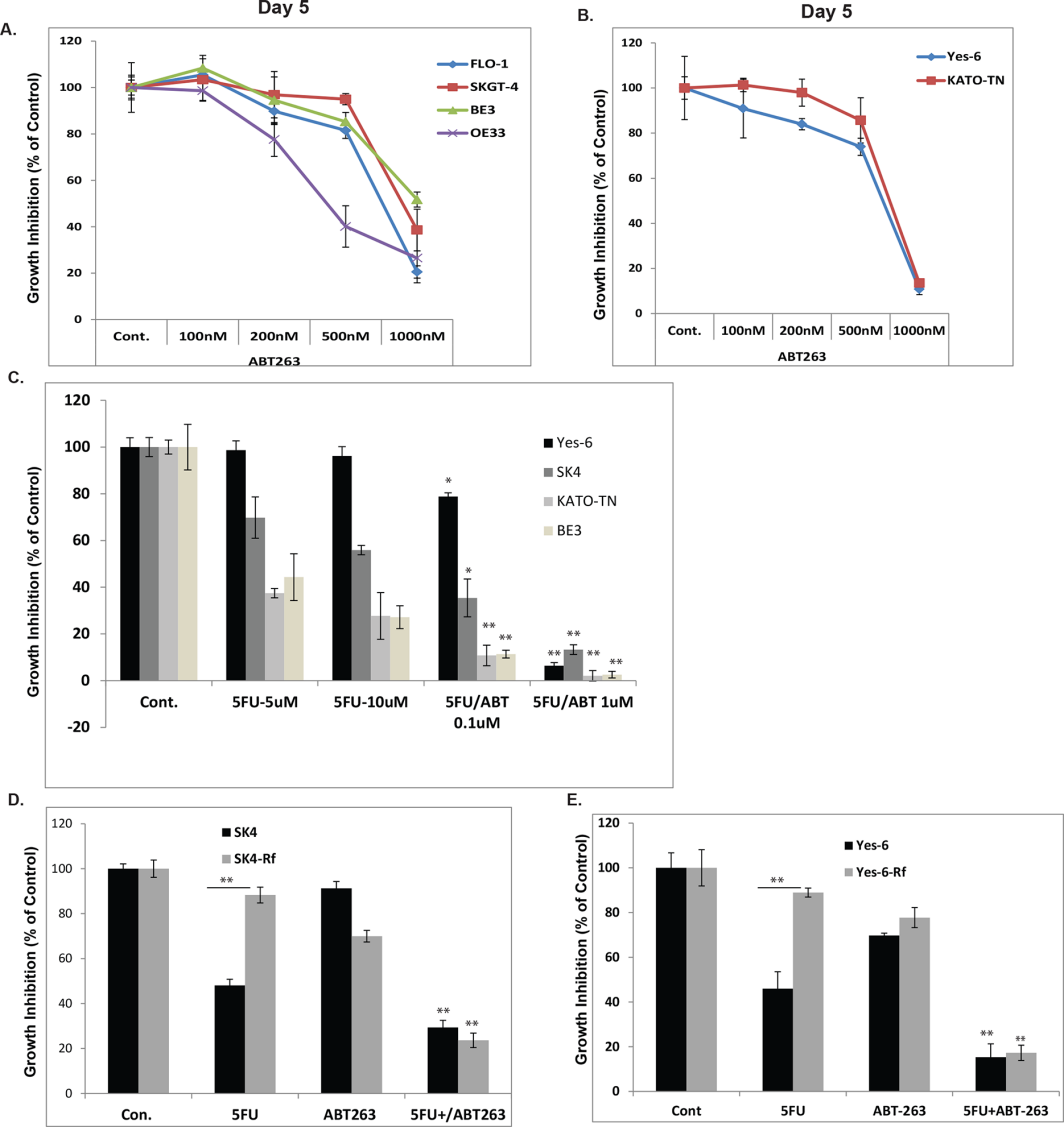
ABT-263 potently inhibit EC cell growth and synergizes with 5-FU on both sensitive and resistant EC cells **A.** & **B.** Four EAC cell lines (left panel) and two ESCC cell lines (right panel) were treated with 0.1% DMSO (as control) or ABT-263 at different dosage as indicated for 5 days, cell growth inhibition was measured using MTS assay and calculated as percent of control. **C.** Four EC cell lines treated with 5-FU at different dosage and in combination with ABT263 at 0.1 μM and 1 μM for 3 days and cell growth inhibition was measured using MTS assay. **D.** SK4 cells and their resistant cells SK4-Rf were treated with 5-FU at 10 μM and ABT-263 at 1 μM either alone or in combination for 3 days, cell growth inhibition was measured using MTS assay. **E.** YES-6 cells and their resistant cells YES-6-Rf were treated with 5-FU at 10 μM and ABT-263 at 1 μM either alone or in combination for three days, cell growth inhibition was measured using MTS assay. **p < 0.01.

**Figure 2:**
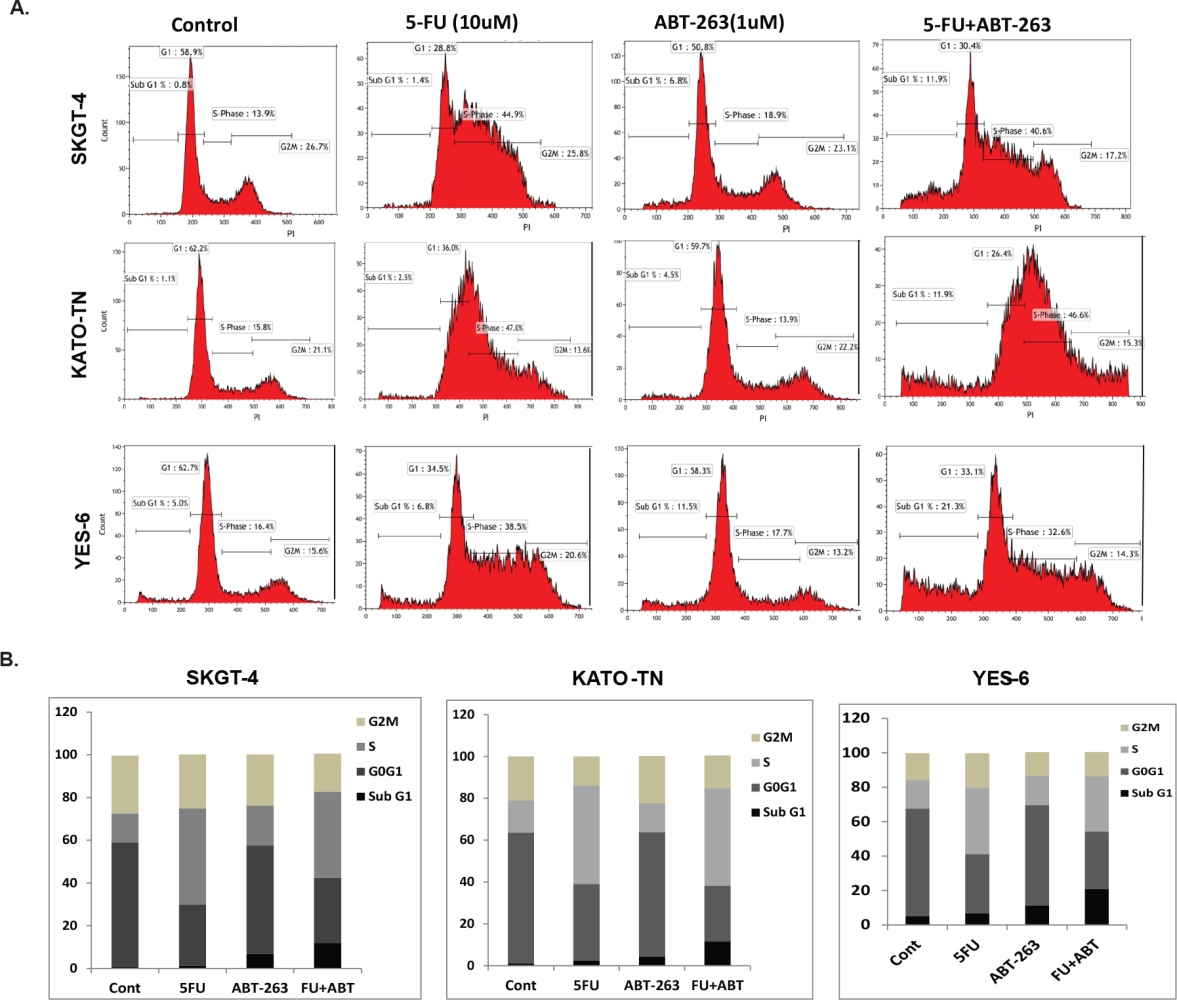
ABT-263 propels the arrested S-phase cells induced by 5-FU into apoptosis **A.** The SKGT-4, KATO-TN and YES-6 cells were seeded onto 6-well plates and treated with 0.1% DMSO (as control) or with ABT-263 1 μM or 5-FU 10 μM or in combination for 48 hours and then fixed and stained for DNA with propidium iodide and then analyzed for DNA histograms and cell cycle phase distribution by flow-cytometry using a FACSCalibur instrument, which showed that 5-FU induced the cells arrested in S-phase and ABT-263 induced the cells arrested in the sub-G1-phase, but the combination resulted in significantly increasing cells in sub-G1 phase. **B.** The cell cycle distribution of SKGT-4, YES-6 and KATO-TN cells were demonstrated in bar graphs according to the proportion of their Sub-G1, G0G1, S and G2M phase after the treatment.

To determine if ABT-263 has potential therapeutic value in EC cell lines, four EC adeno (EAC) cell lines (FLO-1, SKGT-4, BE3 and OE33) and two squamous (ESCC) cell lines (YES-6 and KATO-TN) were treated with ABT-263 at different doses. As indicated in Figures 1A and 2B, ABT263 inhibits both EAC and ESCC cell growth in a dose dependent manner. In relatively low concentrations (<1 μM), ABT263 effectively inhibited cell growth in all cell lines. Most interestingly, when ABT-263 combined with 5-FU, the inhibitory effect was significantly enhanced in six EC cell lines (Figure 1C and Supplementary Figure S3) indicating the synergy between ABT263 and 5-FU.

Chemo-resistance is a major problem in clinical management and overcome chemo-resistance will improve the clinical outcome. Thus, two chemo-resistant cell lines SK4-Rf and Yes-6-Rf were established as described in the Materials & Methods. Next, we sought to determine if ABT-263 can overcome chemo-resistance. As expected, ABT-263 (1 μM) in combination with 5-FU (10 μM) strongly inhibited chemo-resistant cells as well as chemo-sensitive cells; while the single agent, either 5-FU (10 μM) or ABT-263 (1 μM), has minimal effects on these cells (Figure 1D and 1E). This implies that ABT-263 increases the sensitivity of EC resistant cells to 5-FU.

### ABT-263 induces apoptosis that is strongly enhanced by 5-FU in EC cells

To determine whether the growth inhibition observed in EC cells is associated with specific changes in cell cycle distribution, we analyzed the cell cycle using DNA flow cytometry. When SKGT-4, KATO-III, and YES-6 cells were treated with 5-FU, ABT-263, or in combination as indicated dosage for 48 hours, cell cycle phase distributions were analyzed. Results in Figure 2A and 2B show that 5-FU induced cells arrest in S-phase and ABT-263 induced cells in sub-G1 phase. However, the combination of ABT-263 and 5-FU resulted in significant increase in sub-G1 phase (apoptosis) indicating that ABT-263 promotes apoptosis in tumor cells arrested in S-phase (containing DNA damage).

**Figure 3:**
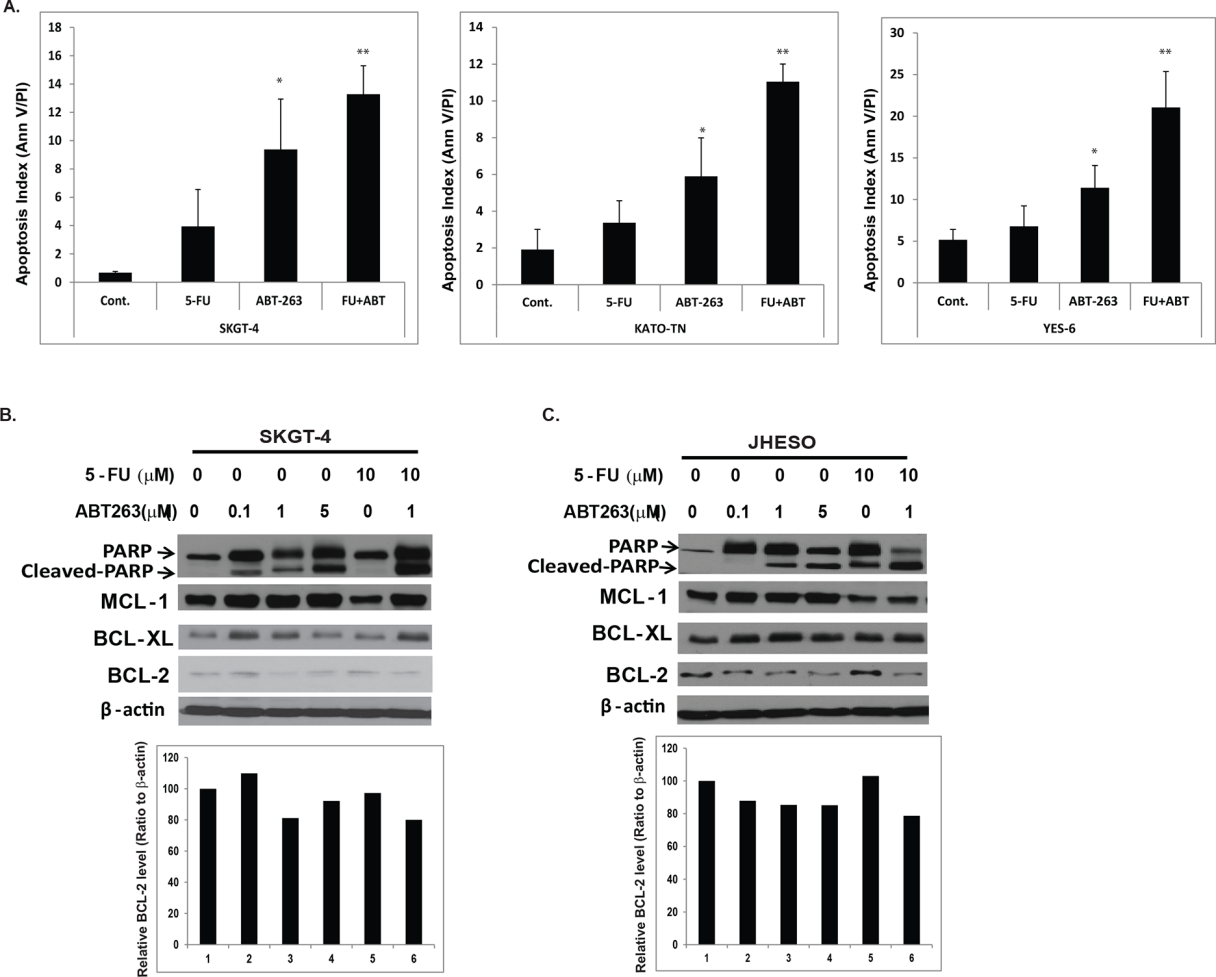
ABT-263 strongly induce apoptosis especially in combination with 5-FU in EC cells **A.** SKGT-4, Yes-6 and KATO-TN cells were treated with 0.1% DMSO (as control) or ABT-263 1 μM or 5-FU 10 μM or in combination and determined the apoptosis index by flow cytometry, which indicated that the apoptosis index were increased, especially in combination treatment cells. **B.** and **C.** Apoptosis associated proteins-PARP, Cleaved PARP and antiapoptotic proteins MCL-1, BCL-2 and BCL-XL were detected using immunoblotting at SKGT-4 and JHESO EC cells treated with 5-FU and ABT263 or in their combination as dosage indicated (Top panel); quantification of BCL-2 expression in SKGT-4 and JHESO EC cells treated with 5-FU and ABT263 or in their combination as dosage indicated was performed using Image J (Lower panel).

To further examine if ABT-263 induces apoptosis in EC cells, we treated SKGT-4, Yes-6 and KATO-TN EC cells with ABT-263, 5-FU, or combination. We observed increase in apoptosis in 2–10 fold by ABT-263 treatment alone. The induction of apoptosis by ABT-263 was significantly amplified by the addition of 5-FU (Figure 3A). Accordingly, cleaved PARP level was strongly increased in the combination treatment group (Figure 3B and 3C top panel) in both SKGT-4 and JHESO EC cells. However, the levels of anti-apoptotic machinery members (BCL-2, BCL-XL and MCL-1) were not dramatically affected (Figure 3B and 3C and Supplementary Figure S1) indicating the strong tumor cell inhibition and induction of apoptosis in EC cells by ABT263 may be due to other mechanisms in addition to its canonical anti-BCL2 family.

### RPPA proteomic array on ABT-263 treated EC cells and analyzed by gene set enriched analysis (GSEA)

**Figure 4:**
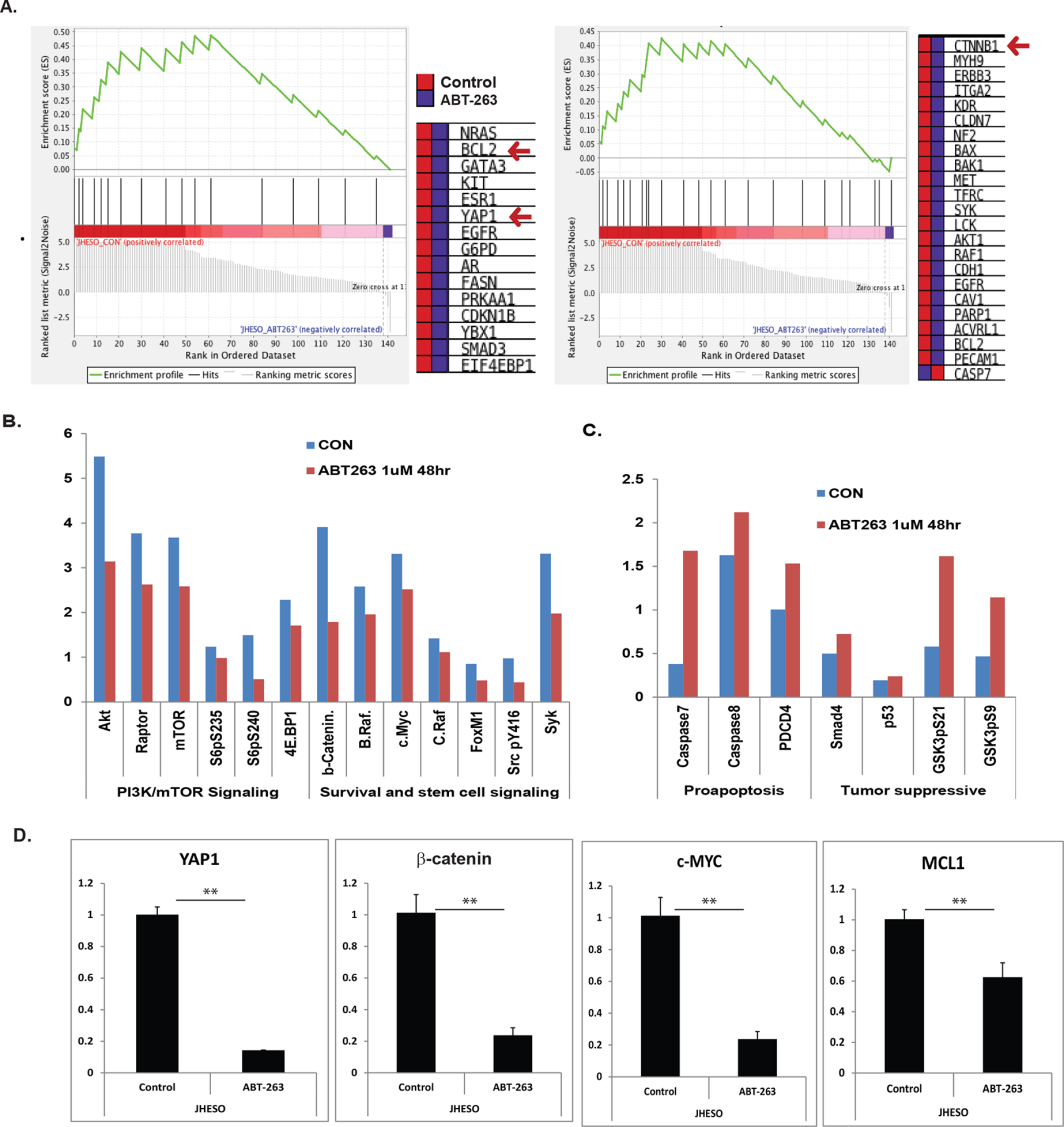
Gene set enriched analysis of RPPA proteomic data on ABT-263 treated JHESO cells and the effects of ABT-263 on cell survival and stemness pathways **A.** Gene set enriched analysis (GSEA) conducted by a specialized bioinformatist (B.L) demonstrated that many genes involving oncogenic (EGFR, PI3K/mTOR) and cancer stemness signaling are down-regulated in ABT-263 (1 μM for 48 hours) treated JHESO cells (Figure 4A). **B.** Down-regulation of genes in PI3K/mTOR and survival and stem cell signaling after normalization by RPPA analysis. **C.** Up-regulation of Genes by ABT-263 in pro-apoptosis and tumor suppression after normalization by RPPA analysis. **D.** Significantly down-regulation of YAP1, β-catenin, c-MYC and MCL-1 by ABT-263 was confirmed using quantitative real-time-PCR. **p < 0.01.

To decipher the novel mechanisms by which ABT-263 enhance the sensitivity of 5-FU in EC cells, we performed RPPA to evaluate 175 proteins expression on EC cells treated with ABT-263 (1 μM) for 48 hours. Gene set enriched analysis (GSEA) conducted by a specialized bioinformatician (B.L), demonstrated that many genes involving oncogenic processes (EGFR, AKT1/2/3 and PI3K/mTOR) and cancer stemness signaling are down-regulated such as β-catenin in Wnt signaling and YAP-1 in Hippo signaling in addition of down-regulation of BCL-2, the major target of ABT-263 (Figure 4A). The quantitative analysis showed a decrease in PI3K/mTOR, survival and stemness signaling (Figure 4B), but the pro-apoptosis and tumor suppressive molecules were up-regulated in ABT-263 treated cells (Figure 4C). Using quantitative real-time PCR, we have confirmed that ABT-263 significantly suppress many important genes that control oncogenic signaling and stemness pathway such as β-catenin; YAP-1, C-MYC and MCL-1 (Figure 4D).

### ABT-263 strongly inhibits expression and activation of Wnt/β-catenin and YAP-1/SOX9 axis in EC cells

**Figure 5:**
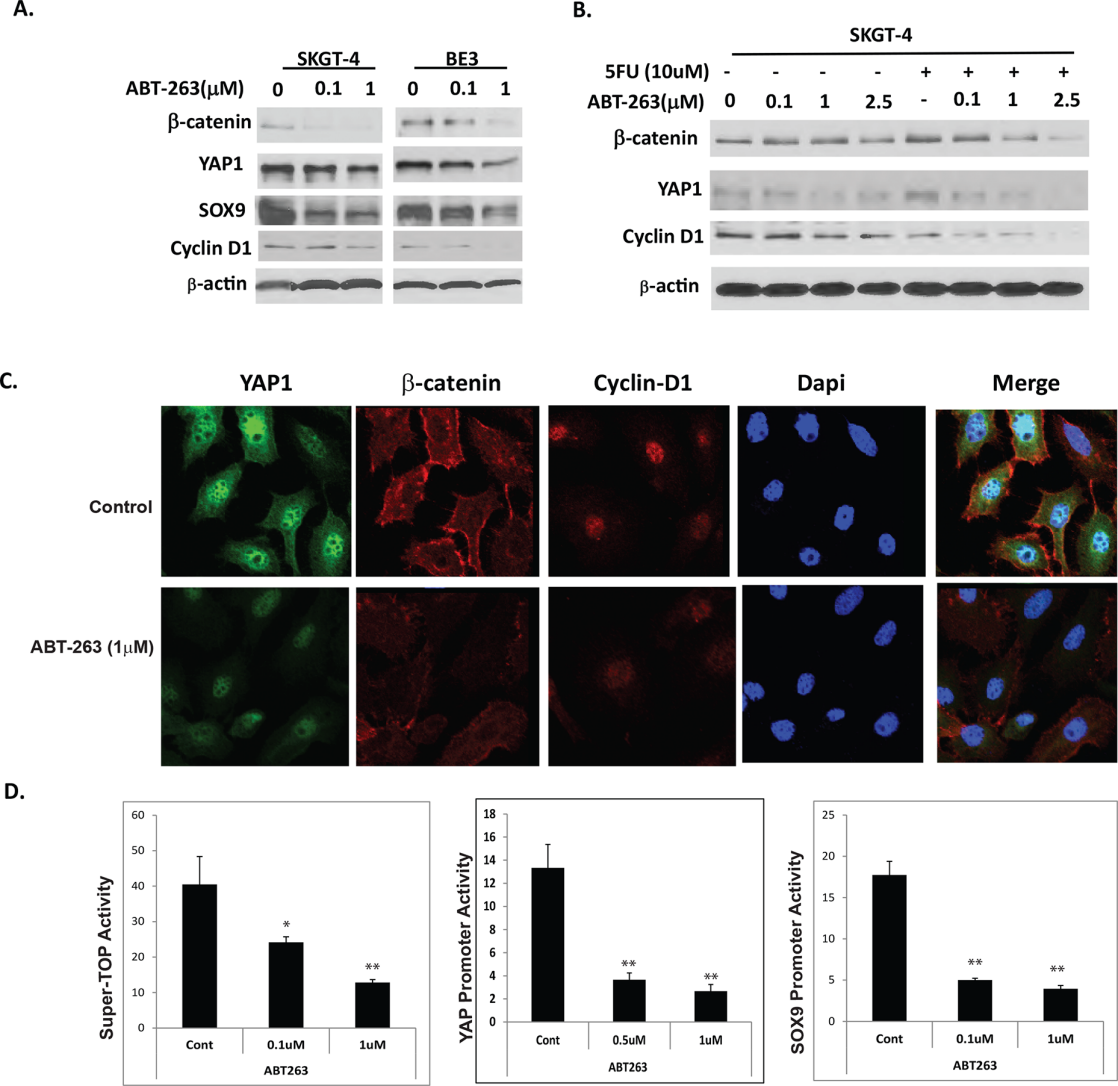
ABT-263 strongly inhibits expression and activation of Wnt/β-catenin and YAP1/SOX9 axes in EC cells **A.** Protein levels of YAP, SOX9, β-catenin and its target Cyclin D1 were determined by immunoblotting in EC cells treated with different dosage of ABT263 for 48 hours. **B.** Protein levels of YAP, β-catenin and its target Cyclin D1 were detected by immunoblotting in SKGT-4 EC cells treated with different dosage of ABT263 and or in combination with 5-FU for 48 hours. **C.** expression level of YAP1, β-catenin and Cyclin D1 was detected by immunofluorescence as described in materials & Methods. **D.** Transient transfection of Super-TOP luciferase (represent Wnt/β-catenin activity) or YAP1 or SOX9 luciferase promoters into SKGT-4 EC cells and treated with ABT263 for 48 hours in different dosage as indicated; Luciferase reporter activities were detected after 48 hours. For all experiments, values shown represent the mean and SD of at least triplicate assays (**p < 0.01).

As stated earlier, ABT-263 was very effective in inhibiting EC cell growth and induced apoptosis especially in combination with 5-FU that is not fully dependent on BCL-2 family proteins (Figure 3). Since stemness pathways such as Wnt/β-catenin and Hippo coactivator YAP-1/SOX9 are central mediators of CSC population maintenance and over growth; and Hippo pathway YAP-1 also mediates anti-apoptotic protein expression and transcription and therapy resistance [34–36]. To further confirm if ABT263 affects CSC signaling, a series of experiments were performed. Results in Figure 5A show that protein levels of β-catenin, its target cyclinD1 and YAP-1 and its target SOX9 decreased in a dose dependent manner when treated with ABT-263 alone (Figure 5A) and the combination with 5-FU. Immunofluorescence (Figure 5C) further confirmed the expression levels of these proteins were decreased by ABT263. To further examine if stemness activity is affected, the SuperTop TCF4 luciferase reporter that reflects the Wnt/β-catenin signaling activity in cells [37] were transfected into SKGT-4 cells and treated with ABT263 at different dosage. Wnt/β-catenin activity decreased in a dose dependent manner with ABT263 exposed for 48 hours. Similarly, co-transfection of Gal4-tead and UAS-Luciferase plasmids which represent YAP-1 activity [38] and SOX9 promoter luciferase [39] respectively into EC cells and treated with ABT-263, YAP-1 and SOX9 activities were suppressed dramatically and were also dose dependent. These data suggest that a novel mechanism of action of ABT263 is on inhibition of stemness pathways-Wnt/β-catenin and YAP-1/SOX9 axis.

### ABT-263 preferentially inhibits tumor sphere formation in ALDH1 positive EC cells

**Figure 6:**
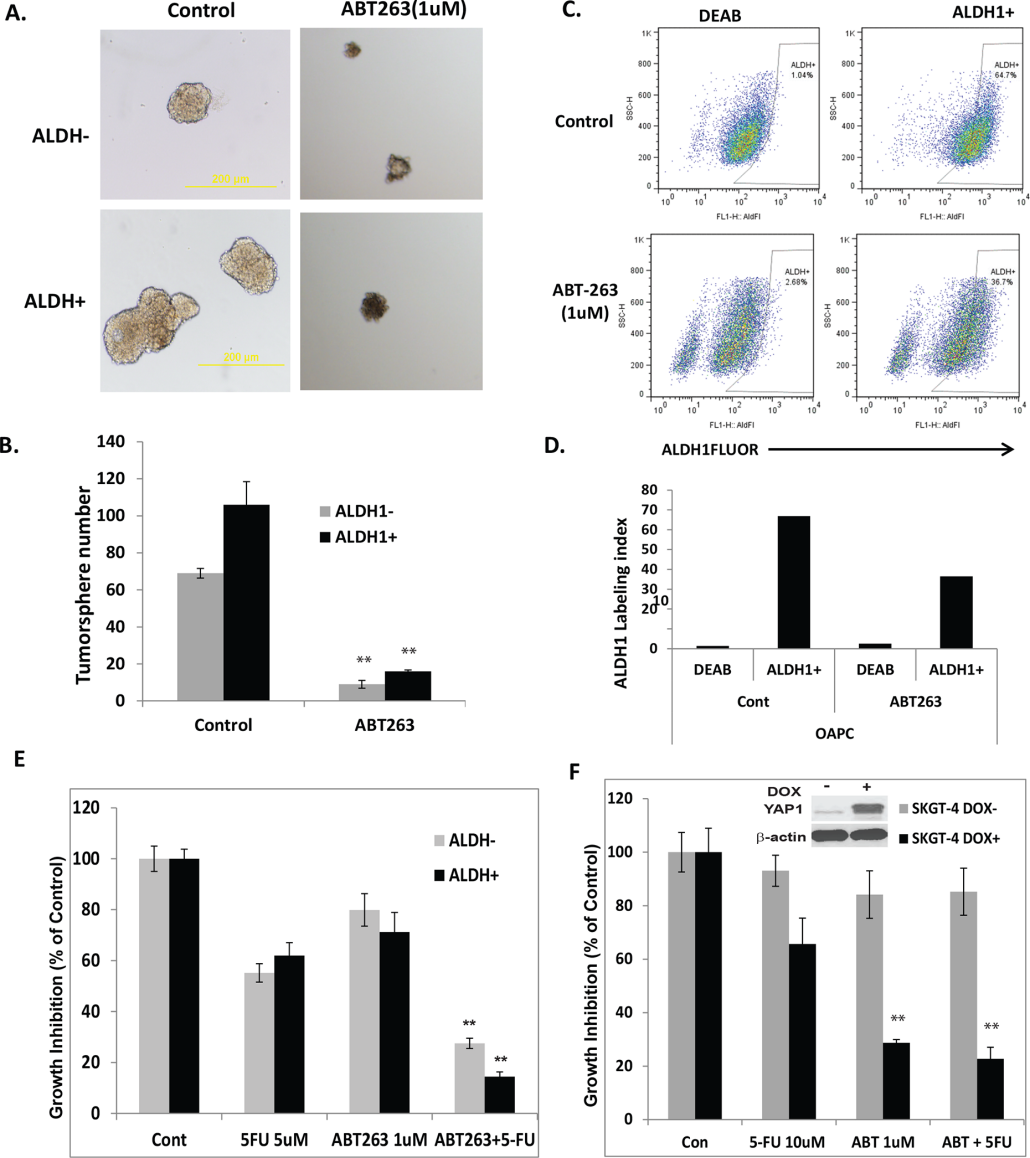
ABT-263 strongly inhibits tumor-sphere formation in both ALDH1+ and ALDH1- EA cells and ABT-263 in combination with 5-FU significantly inhibit ALDH1 positive and induced YAP1 high cell growth **A.** & **B.** ALDH1 positive or negative cells were sorted from JHESO EC cells and tumor sphere assays were done in the sorted cells and add ABT-263 at 1 μM at the beginning of the tumor sphere culture. After 8–10 days of culture, the tumor sphere numbers formed were counted under microscope. Representative fields (A) and the bar graph (B) are demonstrated that ALDH1+ cells formed larger and more tumor spheres than ALDH1- cells, and ABT-263 inhibited the tumor sphere formation in both ALDH1+ and ALDH- cells, but preferentially in the former. **C.** & **D.** JHESO cells were treated with ABT-263 at 1 μM or control for 48 hours and then labeling with ALDH1 antibody that showed ABT-263 reduced the fraction of ALDH1+ cells in the JHESO cells. **E.** ALDH1 positive or negative cells were sorted from JHESO EC cells were treated with 5-FU and ABT-263 either alone or in combination at the concentration indicated for six days, cell growth inhibition was measured using MTS assay. **p < 0.01. **F.** SKGT-4 (PIN20YAP) cells with (DOX+) or without (DOX-) YAP induction by doxycycline and treated with 5-FU and ABT-263 either alone or in combination at the concentration indicated for six days, cell growth inhibition was measured using MTS assay. **p < 0.01.

Having demonstrated down-regulation of CSC signaling components from proteomics assay and confirmed by immunoblotting and immunofluorescence (Figure 5) in ABT-263 treated EC cells, we wondered if ABT-263 could affect the CSC population in EC cells. ALDH1 being a reliable CSC marker [40]. We sorted ALDH1 positive (ALDH1+) or ALDH1 negative (ALDH1-) cells from JHESO cells and assessed their tumor sphere formation capacity with or without ABT-263 treatment at 1 μM for 8–10 days. As shown in Figure 6A, ALDH1+ cells formed larger and more numerous tumor spheres than ALDH1- cells and ABT-263 inhibited the tumor sphere formation in both ALDH1+ and ALDH- cells, but preferentially in the former. Representative fields and the bar graph analysis are shown in Figures 6A and 6B, respectively. In addition, ABT-263 also reduced the fraction of ALDH1+ cells in the JHESO cells (Figures 6C and 6D). This suggests that ABT-263 is very effective on inhibition of CSC population that is at the core of tumor progression, resistance, heteogenecity and metastases.

### ABT-263 in combination of 5-FU preferentially inhibits tumor cell growth in ALDH1 positive and induced YAP1 high EC cells

To further determine the effects of ABT263 alone or in combination with 5-FU on inhibition of ALDH1+ cells, ALDH1+ or ALDH1- cells sorted from JHESO cells were seeded in 96 well and treated with ABT263, 5-FU alone or in combination. Resulted in Figure 6E and Supplementary Figure S2 have shown that the combination of 5-FU and ABT263 led to a significantly decrease in both ALDH1+ and ALDH- cell growth and preferentially inhibition of ALDH1+ cells; while ABT263 or 5-FU alone has minimal effects. Intriguingly, when SKGT4 (PIN20YAP1, an inducible YAP1 construct) cells with (DOX+) or without (DOX-) induction of YAP-1 and treated with ABT263, 5-FU, or in combination, results in Figure 6F demonstrat that ABT263 alone preferentially inhibited YAP-1 high SKGT-4 cells (DOX+) compared to cells without YAP-1 induction (DOX-) and the combination of ABT263 plus 5-FU produced greatest inhibition on YAP-1 induced SKGT-4 cells. Successful induction of YAP-1 by doxycycline is shown in the Figure 6F (inset). Similar results were observed in KATO-TN YAP-1 induced cells. This indicates that ABT263 and its combination with 5-FU preferentially inhibits YAP-1 high cell growth due to the suppression of the YAP-1/SOX9 axis.

### Strong antitumor effect of ABT-263 in combination of 5-FU is through suppression of CSC genes (YAP-1/SOX9) *in vivo*

**Figure 7:**
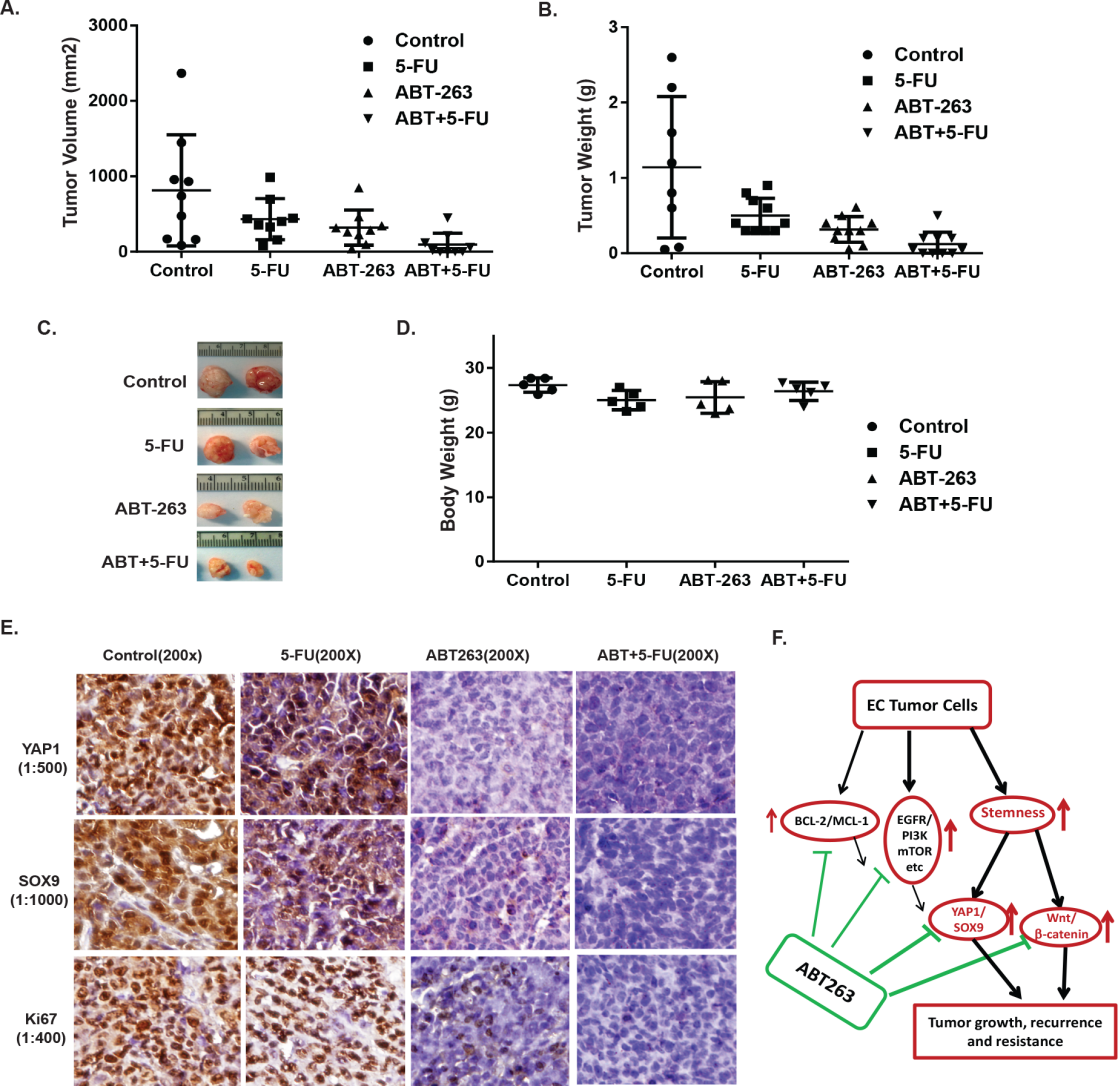
ABT263 in combination with 5-FU strongly inhibit EC tumor growth and suppress expression of stemness genes (YAP1/SOX9) *in vivo* JHESO cells (1.5 × 10^6^) were injected subcutaneously in nude mice, each mouse have two sites (left, right) injections; 5 mice/group and treated with either ABT263 alone, 5-FU alone or in combination as described in Materials & Methods. Tumor Volume **A.** tumor weight **B.** and mouse body weight in each group **D.** were measured and calculated as described in Materials & Methods. Representative tumors **C.** from each group after 4 weeks are shown. Each point represents mean tumor volume/weight and SD from five mice. **E.** Immunohistochemistry for YAP1, SOX9 and Ki67 was performed in mouse tumor tissues derived from JHESO xenograft nude mice. **F.** Proposed model by which effects of ABT263 on EC cell growth and resistance by targeting stemness pathways and oncogenic signaling in addition to its canonical function on BCL-2 inhibition.

Nude mice bearing JHESO cell xenografts were divided randomly into 4 groups and then treated with control (PBS), ABT-263 alone, 5-FU alone and in combination as described in Materials & Methods. At the end of three week dosing schedule, xenograft weight and volume and mice body weight were measured (Figure 7A–7D). Results from *in vivo* experiments demonstrated that mice treated with ABT-263 greatly reduced tumor volume and weight *in vivo*, while the mice treated with ABT-263 in combination with 5-FU, the significant reduction of tumor weights and tumor volumes were observed compared with 5-FU alone (Figure 7A and 7B), while mice body weights did not change significantly. In addition, the level of YAP1, SOX9 and proliferation marker KI67 in mice tumors was dramatically diminished by the combination treatment of ABT-263 and 5-FU (Figure 7E). Thus, ABT-263 in combination of 5-FU has strong antitumor effects *in vivo* and these effects are, at least in part, due to the inhibition of CSC genes-YAP1 and SOX9.

## DISCUSSION

EC clearly presents numerous challenges in the clinic. The degree of patient benefit is limited even after many years of research. New therapeutic targets are needed to improve patient outcome. Our novel data demonstrate that small molecules like ABT-263 are promising and need to be pursued in the clinic. In this study, we show that ABT-263 in combination with 5-FU strongly induces apoptosis and enhances sensitivity to both EAC as well as ESCC; most importantly the combination is lethal to chemo-resistant cells by targeting CSCs population through inhibiting Wnt/β-catenin and or YAP-1/SOX9 axes.

EC cell lines and their resistant counterparts when treated with ABT-263, 5-FU, or both demonstrated that there was some dose-dependent effect of single drugs; but combination considerably reduced cell viability by enhancing apoptosis. 5-FU acting as a thymidylate synthase (TS) inhibitor induces cell arrest in the S-phase and then initiates apoptosis [29], but ABT-263 initiates apoptosis by targeting BCL-2 family proteins through by mimicking the BH3 domain [11]. We confirmed that 5-FU induced cells arrested in S-phase, while in combination with ABT-263, ABT263 propel the S-phase arrested cells induced by 5-FU into apoptosis. ABT-263 with 5-FU at the low dosage had synergetic inhibitory effect on cell proliferation and apoptosis, as confirmed by the increased cleaved PARP proteins by western blot analysis. However, the effect of ABT263 did not fully depend on its canonical targets BCL-2, BCL-XL and BCL-w, since EC cells we tested have either low BCL-2 or no BCL-2 expression (Figure 3B) and BCL-2 expression is often low or absent in EAC tissues and cell lines [41]. Therefore, the observed strong inhibitory effect by ABT263 with 5-FU, especially on EC resistant cells, is likely due to novel mechanism that has not been described.

To explore the potential novel mechanisms of ABT-263 action, we employed RPPA to evaluate 175 proteins expression on JHESO EC cells treated by ABT-263. GSEA analysis our RPPA results show that CSC stemness pathways and PI3K/mTOR pathways were inhibited, while proapoptosis and tumor suppressive genes were induced by ABT-263. More impressively CSCs have the capacity to repopulate tumors and drive malignant progression and mediate radio- and chemoresistance [18] and dysregulation of CSC signaling like Hippo/YAP1, Wnt/β-catenin have been implicated in the maintenance of CSC population and confers therapy resistance [21, 23]. YAP-1 is frequently overexpressed in many types of cancers and mediates constitutive and acquired treatment resistance in EAC cells [36, 42, 43]. In this study, we identified for the first time that ABT263 suppress nuclear expression of YAP-1 and its target SOX9; and inhibits their transcription activity as shown by transfection of YAP-1 and SOX9 promoter activity. Most importantly, ABT-263 alone or in combination of 5-FU demonstrated strong inhibitory effect on expression of YAP-1 and SOX9 *in vivo* xenografts. Similarly, ABT263 was able to decrease β-catenin and its target CyclinD1 expression and their activity reflected by Super-TOP activity. Most importantly, ABT263 reduced CSC population and tumor sphere formation. We show that ABT-263 could reduce the CSC population, as noted ABT263 preferentially inhibits ALDH1 positive EC cells tumor sphere formation and decrease the proportion of ALDH1 positive cell population. It is reported that ALDH1, YAP-1/SOX-9 and β-Catenin, as reliable CSC markers (35–40) are either associated with resistance or mediate resistance. Our study showed that ABT-263 with 5-FU strongly inhibits ALDH1 positive EAC cell growth (Figure 6E). Further, when EC cells have high levels of YAP1 expression by doxycycline induction (DOX+), the combination treatment of ABT-263 and 5-FU significantly inhibits induced YAP1 high EC cell growth *in vitro*. This indicates that ABT-263 strongly induces EC cells apoptosis and sensitizes cells to 5-FU through targeting CSC population by inhibiting both YAP-1/SOX9 axis and Wnt/β-catenin signaling.

In conclusion, our data show that ABT-263 can induce apoptosis, inhibit EC cell growth, and overcome resistance in EC cells. However, this effect is greatly amplified in combination with 5-FU. The synergistic effects of ABT-263 and 5-FU rely on inhibiting CSC signaling components mainly on YAP-1/SOX9 axis and Wnt/β-catenin signaling in addition to its canonical targets (BCL-2) (Figure 7E). Therefore, our unique data indicate that the combination regimens of ABT-263 and 5-FU appear promising as therapeutic option and need to be pursued in the clinics.

## MATERIALS AND METHODS

### Cells and reagents

The human esophageal adenocarcinoma (EAC) cell lines BE3, SKGT-4 (SK4), JHESO have been previously described [31, 39]. The human esophageal squamous cell carcinoma (ESCC) cell lines Yes-6 and KATO-TN were kindly provided by Dr. Health Skinner (UT M.D. Anderson Cancer Center). To establish 5-FU-resistant subclones, Yes-6 and SKGT-4 parent EC cells were cultured with various concentrations of 5-FU for 3–5 weeks, and the surviving cells were collected. This collection procedure was repeated four times. The establishment of these 5-FU-resistant subclones took 3–6 months and newly derived 5-FU-resistant clones, designated as Yes-6-Rf and SK4-Rf. These cells were authorized and re-characterized in the characterized cell line core facility of UT MD Anderson Cancer Center every 6 months. ABT-263 was obtained from Calbiochem (San Diego, CA). 5-FU was from Sigma (Saint Louis, MO). Antibodies β-catenin, MCL-1, BCL2, BCL-XL and PARP were purchased from Cell Signaling (Beverly, MA). SOX9 was purchased from Chemicon (Millipore, Billerica, MA).

### Cell proliferation assay

The EC cells and their resistant counterparts were treated with 0.1% DMSO (as control), ABT-263 or 5-FU or combination at different dosage for 6 days as indicated and then the cell viability were detected using MTS assay as following: cell proliferation assays were performed using the CellTiter 96 aqueous nonradioactive cell proliferation assay (MTS) according to the instructions of the manufacturer (Promega Co., Madison, WI). Similarly, Sorted ALDH1 positive vs negative cells from JHESO cells and doxycycline induced YAP1 high SKGT-4 (DOX+) vs YAP1 low cells (SKGT-4 DOX-) [20] were treated with 0.1% DMSO (as control), ABT-263 or 5-FU or combination at different dosage for 6 days and then the cell viability were detected using MTS assay as above described. All assays were performed in triplicate and repeated at least three times.

### Flow cytometric and apoptotic analysis

Apoptotic analysis by flow cytometry was performed as previously described [44]. In brief, SKGT-4, KATO-TN and Yes-6 cells were seeded onto six-well plates (1 × 10^5^ per well) in DMEM and cultured for 24 h to allow cell attachment. The cells were then treated with 0.1% DMSO (as control) or ABT-263 and/or 5-FU at different dosage for 48 h. Then the cells were harvested, fixed with methanol, washed, treated with RNase A, and stained for DNA with propidium iodide (Sigma, Saint Louis, MO) and then were analyzed for DNA histograms and cell cycle phase distribution by flow cytometry using a FACSCalibur instrument (Becton Dickinson, NC).

### Flow cytometric labeling and fluorescence-activated cell sorting

ALDH1 activity was assessed by fluorescence-activated cell sorting in EC cell line JHESO according to the ALDEFLUOR based cell detection kit (STEMCELL technologies Inc, Vancouver, BC, V5Z 1B3, Canada) following the protocol and Diethylaminobenzaldehyde (DEAB) was used to inhibit ALDH1 activity to show the specificity of the detection. JHESO cells were treated with ABT-263 at 1 μM or control for 48 hours and then labeling with ALDH1 antibody. The ALDH1 positive (ALDH1+) or negative (ALDH1-) cells were sorted by fluorescence-activated cell sorting according to the ALDEFLUOR detection kit [40]. ALDEFLUOR/DEAB treated cells were used to define negative gates. FACS was performed with >1 × 10^6^ cells using the BD FACSCanto II or FACSAria (Becton Dickinson, NC).

### Tumor sphere formation assay

Sphere culture was performed as previously described [20]. Briefly, FACS-isolated ALDH1+ or ALDH1- cell populations (1000/well) were seeded in triplicate onto a 6-well ultra-low attachment plate. After 10–14 days of culture, the number of tumor spheres formed (diameter > 100 μm) was counted under microscope.

### Protein extraction and Western blot analysis

Protein isolation and Western blot analyses were performed as previously described [20].

### Indirect immunofluorescence

Indirect immunofluorescence staining was performed as described (20). Expression and localization of YAP1, b-catenin and CyclinD1 were observed under a confocal microscope system (FluoView FV500; Olympus, Melville, NY) and analyzed by CellQuest PRO software (BD Biosciences).

### Reverse-phase protein arrays (RPPA)

RPPA analysis was performed in cell lysate from JHESO cells control and treated with ABT-263 at 1 μM for 48 h in RPPA core facility, the U.T. M.D. Anderson Cancer Center. Samples were serially diluted 2-fold for 5 dilutions and probed with 175 antibodies and arrayed on nitrocellulose-coated slides. Relative protein levels were normalized for protein loading and determined by interpolation of each dilution curve from the standard curve as previously described [45].

### Transient transfection, and luciferase reporter assays

Super-Top TCF4 luciferase reporter plasmid under the control of eight TCF4 consensus was provided by Dr. C. Liu (The University of Kentucky, KY). Gal4-tead and UAS-Luciferase plasmids which represent YAP1 activity [38] were provided by Dr. R J Johnson (U.T.MD Anderson Cancer Center, TX). The SOX9 luciferase reporter was previously described [39]. Transient co-transfection and activity assay of above luciferase reporters and Renilla vector respectively were performed as previously [39].

### *In vivo* xenograft mouse model

JHESO cells were subcutaneously injected with 2 × 10^6^ cells in nude mice. *n* = 5 each group. After around 10 days, ABT263 was applied by oral gauge, 50 mg/kg/mouse, 5-FU was applied by intra-peritoneal (IP), 30 mg/kg/mouse and their combination, three times a week for total two weeks. Control group was applied same volume of PBS (100 μl/mouse). The tumor volume, tumor weight and mouse body weight were measured as previously [28]. All the measurements were compared using unpaired Student's *t*-test.

### Statistical analysis

Data were analyzed using the student *t*-test; A *P* value of < 0.05 was required for statistical significance, and all tests were two-sided. All tests were done with SPSS 10.1 software (SPSS, Inc., Chicago, IL).

## SUPPLEMENTARY FIGURES


