# Supplementary material for: ABT-263 induces apoptosis and synergizes with chemotherapy by targeting stemness pathways in esophageal cancer
Source: Oncotarget. 2015 Jul 17;6(28):25883–96. doi: 10.18632/oncotarget.4540 (PMC4694873; doi:10.18632/oncotarget.4540)
Supplement: Supplementary file 1 [file oncotarget-06-25883-s001.pdf]

SUPPLEMENTARY FIGURES

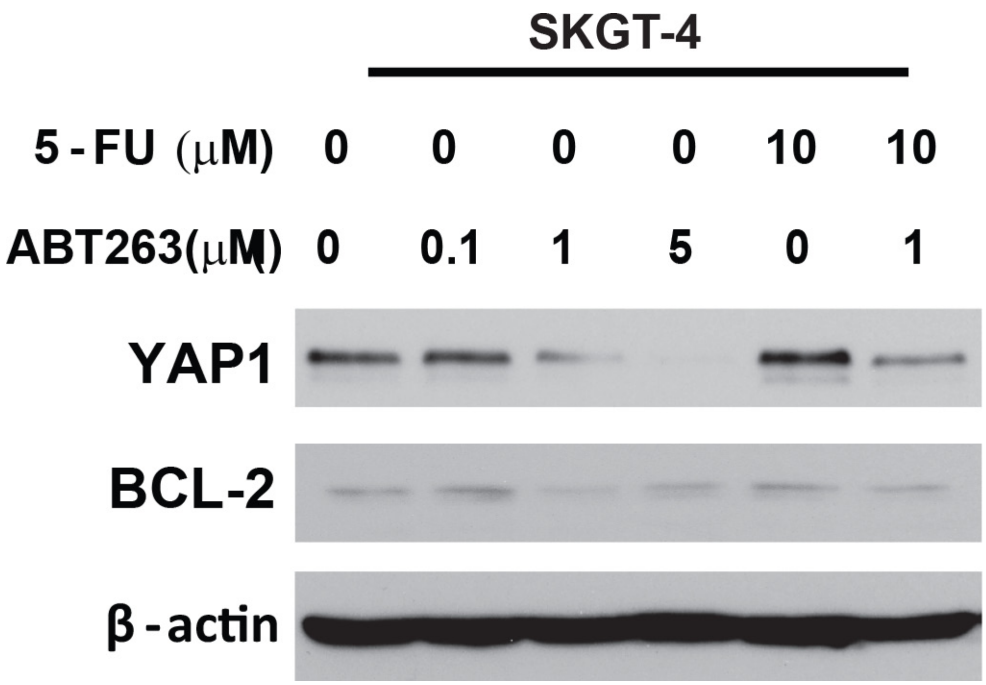

Supplementary Figure S1: ABT-263 significantly suppress YAP1 expression. YAP1 and BCL-2 were detected using immunoblotting at SKGT-4 and JHESO EC cells treated with 5-FU and ABT263 or in their combination as dosage indicated.

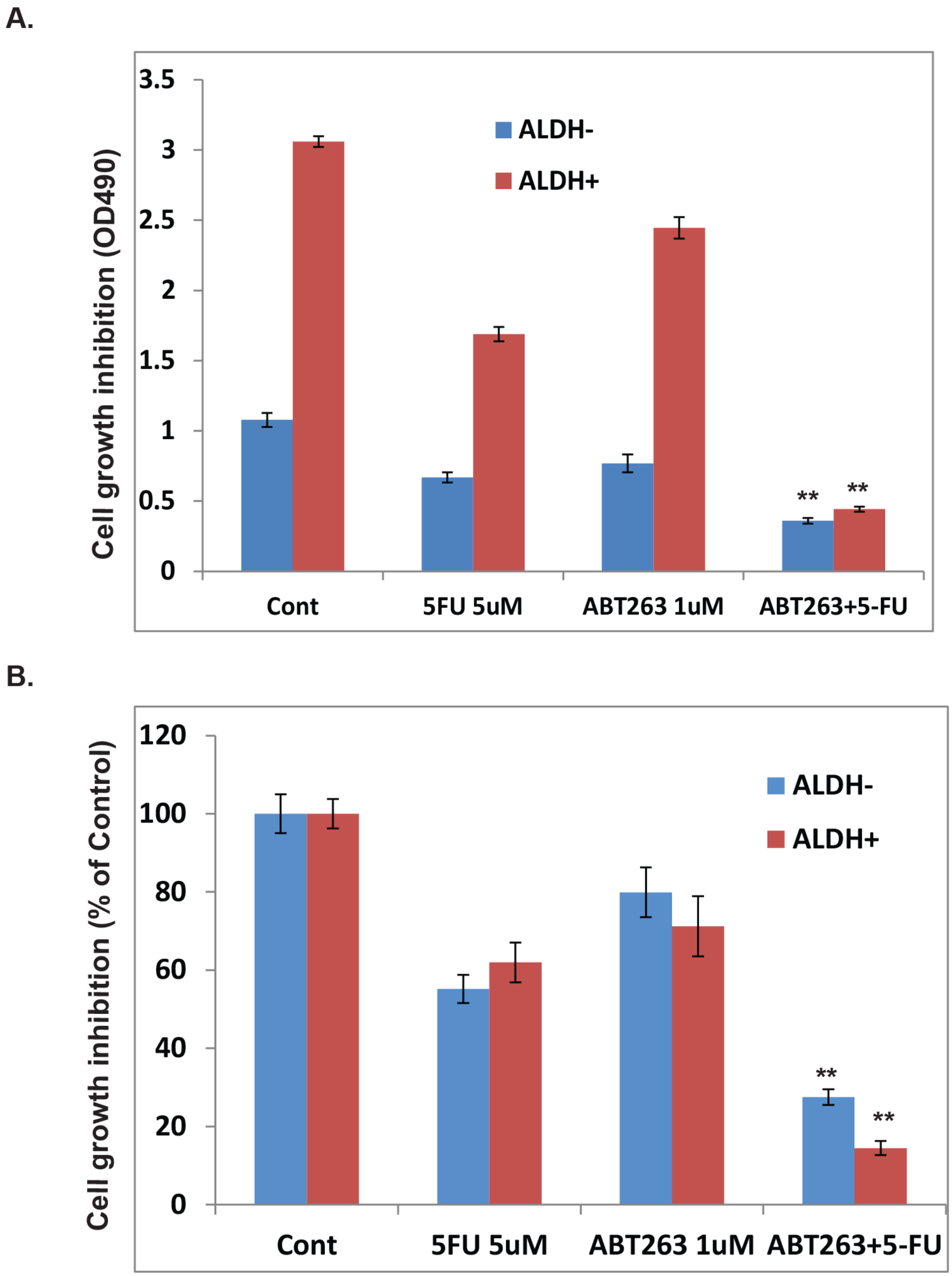

Supplementary Figure S2: The combination of ABT-263 and 5-FU preferentially inhibit ALDH1 positive EC cells. ALDH1 positive or negative cells were sorted from JHESO EC cells were treated with 5-FU and ABT-263 either alone or in combination at the concentration indicated for six days, cell growth inhibition was measured using MTS assay. $**p < 0.01$. **A.** Graph made is based on OD490; **B.** Graph made is based on the percent of the control for each treated group. $**p < 0.01$.

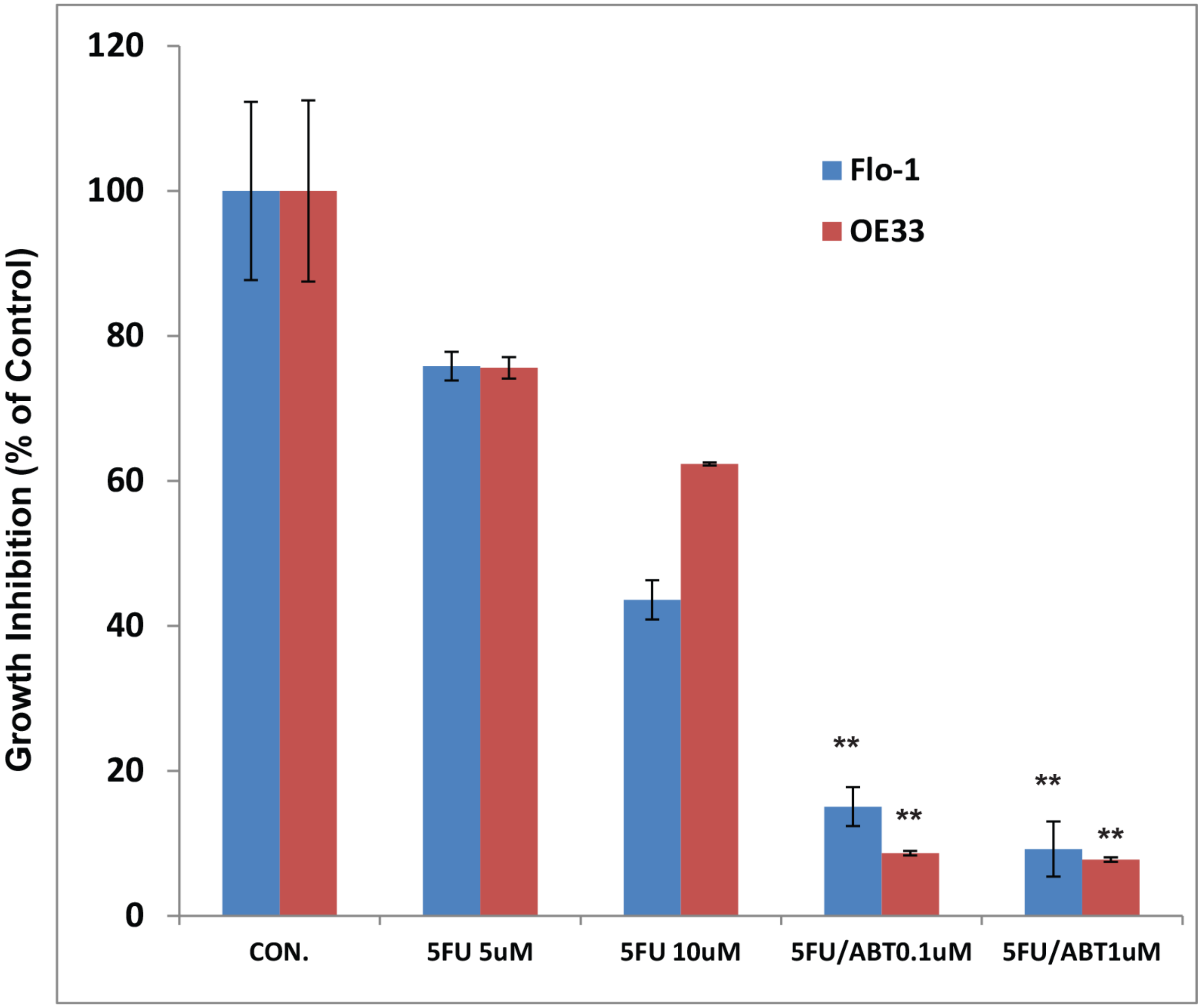

Supplementary Figure S3: ABT-263 potently inhibit tumor cell growth and synergizes with 5-FU in Flo-1 and OE33 EC cells. Two additional EC cell lines-Flo-1 and OE33 treated with 5-FU at different dosage and in combination with ABT263 at 0.1 uM and 1 uM for 3 days and cell growth inhibition was measured using MTS assay. ** $p < 0.001$.
